# Design and Printing of a Low-Cost 3D-Printed Nasal Osteotomy Training Model: Development and Feasibility Study

**DOI:** 10.2196/19792

**Published:** 2020-11-17

**Authors:** Michelle Ho, Jared Goldfarb, Roxana Moayer, Uche Nwagu, Rohan Ganti, Howard Krein, Ryan Heffelfinger, Morgan Leigh Hutchinson

**Affiliations:** 1 Health Design Lab Thomas Jefferson University Philadelphia, PA United States; 2 Department of Otolaryngology Thomas Jefferson University Philadelphia, PA United States

**Keywords:** 3D printing, nasal osteotomy, simulation, education, low-cost

## Abstract

**Background:**

Nasal osteotomy is a commonly performed procedure during rhinoplasty for both functional and cosmetic reasons. Teaching and learning this procedure proves difficult due to the reliance on nuanced tactile feedback. For surgical simulation, trainees are traditionally limited to cadaveric bones, which can be costly and difficult to obtain.

**Objective:**

This study aimed to design and print a low-cost midface model for nasal osteotomy simulation.

**Methods:**

A 3D reconstruction of the midface was modified using the free open-source design software Meshmixer (Autodesk Inc). The pyriform aperture was smoothed, and support rods were added to hold the fragments generated from the simulation in place. Several models with various infill densities were printed using a desktop 3D printer to determine which model best mimicked human facial bone.

**Results:**

A midface simulation set was designed using a desktop 3D printer, polylactic acid filament, and easily accessible tools. A nasal osteotomy procedure was successfully simulated using the model.

**Conclusions:**

3D printing is a low-cost, accessible technology that can be used to create simulation models. With growing restrictions on trainee duty hours, the simulation set can be used by programs to augment surgical training.

## Introduction

### Background

The use of simulation is increasing in postgraduate medical education. Driving this change is the need to expose residents to procedures within the confines of resident duty hours and attention to patient safety. The benefits of simulation have been reported widely in the literature. Systematic reviews and meta-analyses have reported that simulation training is associated with positive outcomes, such as knowledge and procedural skills [1,2].

Traditionally, cadaveric bones are used by surgical residents for simulation to learn about anatomy and surgical techniques. Benefits of cadaveric bones include high fidelity to in vivo anatomy and opportunity for simulation with tactile feedback. Drawbacks, however, include limited supply, high cost, and lack of pathology [3]. The use of virtual reality (VR) simulators is also growing. In their review of VR training in laparoscopic surgery, Alaker et al [4] suggested that VR in combination with haptic feedback is the most effective way to deliver VR training. Similar to cadaveric models, however, high cost of acquisition can be a barrier to utilizing VR [5].

Within medicine, advances in technology and affordability have expanded the use of 3D models. This technology utilizes postprocessing of computed tomography (CT) and magnetic resonance imaging (MRI) data coupled with 3D printers to create unique models that are used for patient education, presurgical planning, and trainee education. Due to the complexity of procedures and similarity of bones to 3D printing material, facial plastics and otolaryngology simulators have been widely explored. VanKoevering and Malloy [6] reported a variety of simulators, including auricular reconstruction, endoscopic endonasal skull base drilling, and laryngeal simulators. Previously, Zabaneh et al [7] reported the design and fabrication of a training model for rhinoplasty simulation. This model used various molds to simulate tissue and skin layers and was printed in acrylonitrile butadiene styrene (ABS) on an inkjet 3D printer, significantly increasing the cost and accessibility of the model.

Rhinoplasty is among the most commonly performed facial plastic procedures in the United States and one of the most challenging [8,9]. During a rhinoplasty, nasal osteotomies—which involve applying high force energy to cut into a bone using osteotomes—may be performed to straighten the nasal vault to improve cosmesis and correct nasal obstruction. In a rhinoplasty procedure, nasal bone osteotomy is a particularly challenging and potentially dangerous maneuver [8]. The procedure relies largely on tactile feedback rather than direct visualization; therefore, this procedure is difficult to teach and learn.

### Objective

The objective of this study was to develop an accessible, low-cost 3D training model for nasal osteotomy.

## Methods

### Image Segmentation

**Figure 1:**
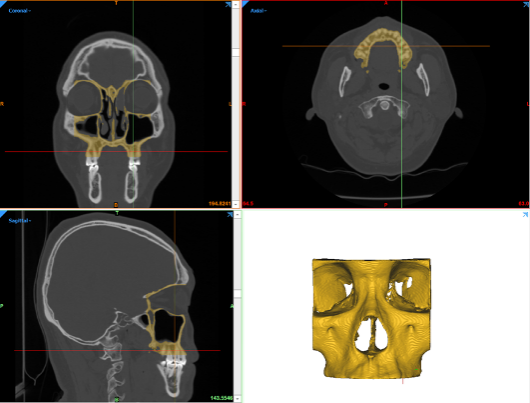
Thresholding and segmentation of midface.

Routine diagnostic CT imaging was obtained from patients undergoing treatment of head and neck malignancy under a protocol approved by the institutional review board at Thomas Jefferson University. Original image data, in the file format of digital imaging and communications in medicine (DICOM), were reviewed by otolaryngologists to identify the presence of suitable anatomic features, regions of interest, and absence of dental artifacts. Imaging was performed using a LightSpeed Pro(16) CT scanner (GE Medical Systems) at 0.625 mm. The DICOM image data were subsequently deidentified and imported into processing software (Mimics Innovation Suite, Materialise NV). The data were processed to reduce image noise, and thresholding was used to isolate the midface (Figure 1).

**Figure 2:**
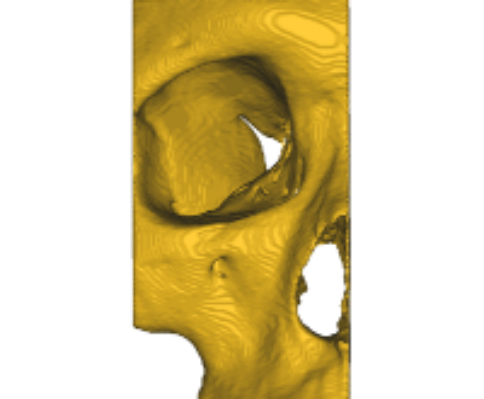
Cropping of midface.

The software was then used to create a 3D reconstruction of the midface. Using the cropping tool, the midface was then split at the middle of the nasal septum (Figure 2). After segmentation and cropping, the model was exported as a surface tessellation language (STL) file.

### Design

**Figure 3:**
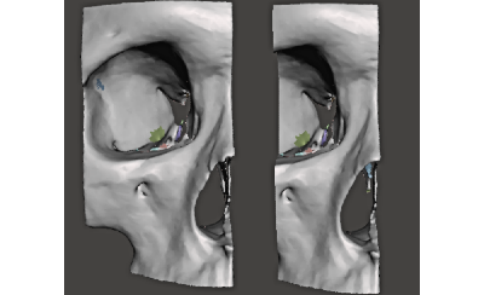
Anterior view of midface before and after smoothing of nasal prominence.

The STL file was imported into the open-source software Meshmixer (Autodesk Inc) for postprocessing, design, and repair of mesh surface for printability. Using the sculpt brush tools, the nasal pyriform aperture was smoothed (Figure 3).

**Figure 4:**
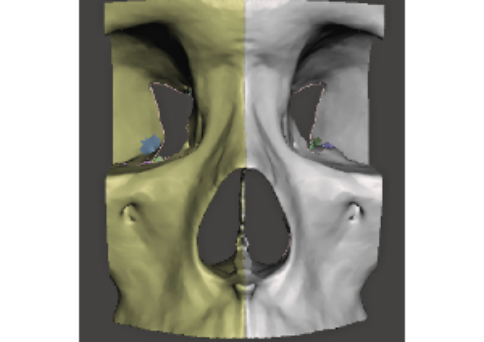
Original (yellow) and mirrored (silver) midface model.

Additionally, internal bones from the frontal and sphenoid bones (that were not adjacent to the nasal prominence) were removed. The smoothed model was then mirrored to create a symmetrical midface model (Figure 4).

**Figure 5:**
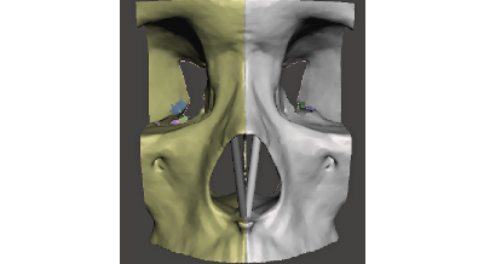
Addition of bilateral support rods.

Bilateral support rods (3 mm diameter) extending from the base of the nasal spine to the deep aspect of the nasal bones were added to the model to mimic the support normally provided by soft tissue during a nasal osteotomy (Figure 5).

### Printing

The STL file of the midface model was uploaded to Ultimaker Cura (Ultimaker), an open-source 3D printer slicing application, for preprinting, processing, and generation of a UFP file. The following parameters were set in the Ultimaker Cura application: 0.4 mm printer nozzle and layer height of 0.04 mm. Models were printed with one of the following infill densities: 5%, 10%, 15%, 20%, 50%, and 80%. All models were printed using fused deposition modeling (FDM) on an Ultimaker S5 3D printer (Ultimaker) with polylactic acid (PLA) filament and polyvinyl acetate (PVA) filament for supports.

### Assembly

**Figure 6:**
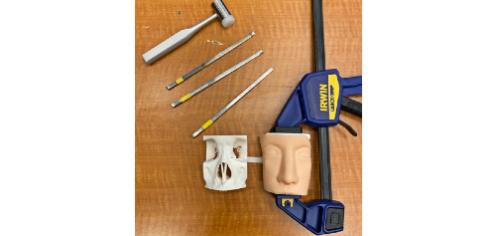
Simulation set with tools, model, and accessories.

To mimic the skin surface, a training tattoo skin mask was cut and placed over the midface model. The mask was secured to the model using Velcro ties. The model was held in place using a 12-inch bar clamp (Figure 6).

## Results

### Printing the Model

A total of 6 models with different infill densities were printed. Each model used approximately 55 g of PLA filament and 54 g of PVA filament. The total printing time was approximately 18 hours for each model. After printing, each model was submerged in tap water until the PVA support material was completely dissolved (approximately 12 hours). Total cost for 1 simulation set was approximately US $37.49 (PLA filament: US $3.85, PVA filament: US $8.10, mask: US $8.99, and bar clamp: US $16.55) [10,11].

### Evaluation of Models

**Figure 7:**
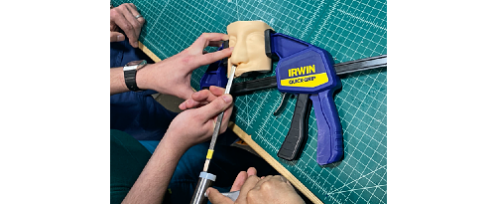
Use of simulation set.

The model was evaluated by 2 attending facial plastic surgeons and 1 facial plastic surgery fellow to determine its accuracy in simulating human facial bones. The evaluators used osteotomes and hammers to simulate a nasal osteotomy procedure (Figure 7). All evaluators “strongly agreed” that the model with 10% infill density mimicked human bone better than the models with other infill densities.

## Discussion

### Principal Findings

To our knowledge, this is the first reported nasal model for rhinoplasty simulation that is printed on a desktop 3D printer. Osteotomies are considered by many to be a complex surgical technique. As a result, residents and other trainees often have limited opportunities to perform the technique intraoperatively. In this study, our objective was to develop a low-cost, accessible model for trainees to simulate nasal osteotomies.

3D printing is an innovative technology that allows for rapid prototyping of ideas. Moreover, a variety of materials can be printed in different colors, densities, and specifications to simulate an anatomical equivalent. In this study, we used FDM technology and a dual extruder 3D printer. Among 3D printing technology, FDM is the most widespread technique, and it is also cost-effective [12]. PLA was chosen for the model, as it is one of the most popular materials for 3D printing and is biocompatible, nontoxic, and biodegradable [13]. Use of support material was necessary to print overhangs, intricate details, and internal cavities (within and surrounding the nasal cavity) that would otherwise be impossible to print due to gravity. PVA was chosen over other support materials, as it completely dissolves away when submerged in water and leaves behind a smooth surface.

The cost per simulation model was US $11.95, and the simulation accessories cost US $25.54. The model was printed using a desktop dual extruder FDM 3D printer. This type of printer is available at prices starting at US $600. Thus, compared to existing resources, this simulation model is low-cost and accessible, especially for residency training programs that already have access to 3D printing machines.

### Limitations

The 3D-printed midface model was used for surgical simulation and education for otolaryngology residents. However, a few limitations were noted during the production and use of this model. As no objective tool exists to evaluate the fidelity of 3D-printed models for surgical simulation, the team relied on the expertise and experience of facial plastic surgeons to determine which model provided the best simulation experience. During simulation, some users noted that the model appeared to delaminate between the printed layers instead of in the direction of force. Finally, since this model uses forceps to hold the model in place, at least 3 people are needed for each simulation. However, given the limited number of available surgical tool sets, working in groups did not increase the simulation time. Additionally, group members were able to observe and provide feedback to each other.

### Future Direction

Given the rapid advancement of technology in 3D printing, many potential improvements can be made in the model described in this study. In this iteration, the study team focused on determining the infill density that would most closely mimic facial bones. In future studies, other parameters, such as layer height and shell thickness, can be assessed. Blinded comparison will also be used to evaluate the 3D-printed models against other types of simulation models. Finally, in this study, we utilized FDM technology to print our model. In the future, we plan to print our model using different technologies, such as stereolithography (SLA) and material jetting 3D printers, and assess their fidelity to facial bone. In contrast to FDM printing, SLA and material jetting technology use ultraviolet radiation to cure resins into 3D models. SLA printers use an open pool of liquid resin to print models, while material jetting printers use a print head to deposit liquid resin onto a built platform. Additionally, SLA and material jetting 3D printers can print thinner layers (up to 25 microns and 16 microns, respectively) compared to FDM printing. These qualities may allow the models to more closely mimic facial bones.

### Conclusion

In this study, we demonstrated the feasibility of designing and printing a midface model for simulation of medial and lateral osteotomy for rhinoplasty surgery. For residency training programs with access to a 3D printer, this low-cost model can be used for surgical education and simulation.

## Abbreviations

ABS: acrylonitrile butadiene styrene
CT: computed tomography
DICOM: digital imaging and communications in medicine
FDM: fused deposition modeling
MRI: magnetic resonance imaging
PLA: polylactic acid
PVA: polyvinyl acetate
SLA: stereolithography
STL: surface tessellation language
VR: virtual reality
